# Transcriptome Profiling of the Retained Fetal Membranes—An Insight in the Possible Pathogenesis of the Disease

**DOI:** 10.3390/ani11030675

**Published:** 2021-03-03

**Authors:** Joanna Jaworska, Katarzyna Ropka-Molik, Katarzyna Piórkowska, Tomasz Szmatoła, Ilona Kowalczyk-Zięba, Izabela Wocławek-Potocka, Marta Siemieniuch

**Affiliations:** 1 Department of Gamete and Embryo Biology, Institute of Animal Reproduction and Food Research, Polish Academy of Sciences, 10-747 Olsztyn, Poland; i.kowalczyk@pan.olsztyn.pl (I.K.-Z.); i.woclawek-potocka@pan.olsztyn.pl (I.W.-P.); 2 Department of Animal Molecular Biology, National Research Institute of Animal Production, 32-083 Balice, Poland; katarzyna.ropka@izoo.krakow.pl (K.R.-M.); katarzyna.piorkowska@izoo.krakow.pl (K.P.); tomasz.szmatola@izoo.krakow.pl (T.S.); 3 University Centre of Veterinary Medicine Krakow, University of Agriculture in Krakow, Al. Mickiewicza 24/28, 30-059 Krakow, Poland; 4 Research Station of the Institute of Reproduction and Food Research, Polish Academy of Sciences in Popielno, 12-220 Ruciane-Nida, Poland; m.siemieniuch@pan.olsztyn.pl

**Keywords:** placenta, horse, parturition, reproduction

## Abstract

**Simple Summary:**

Retained fetal membranes (RFM) in mares is a disease of a multifactorial etiology with not fully understood pathogenesis. Profound analysis of genes expressed in the placenta may reveal pathways and processes which might be comprised in mares with this disease and hence help to explain the pathogenesis of RFM. This work employed RNA sequencing to identify and compare genes differentially expressed (DEGs) in the placenta of mares that retained fetal membranes and those that released them physiologically. Results showed that within DEGs genes important for apoptosis, inflammatory-related processes, and metabolism of extracellular matrix were identified.

**Abstract:**

Retained fetal membranes (RFM) is one of the most common post-partum diseases of a complex etiology. Moreover, its pathogenesis is still not elucidated. Detailed transcriptomic analysis of physiological and retained placenta may bring profound insight in the pathogenesis of the disease. The aim of the study was to compare the transcriptome of the retained and physiologically released placenta as well as biological pathways and processes in order to determine the possible pathogenesis of the disease. Samples of the endometrium and the allantochorion were taken within 2 h after parturition from control mares (*n* = 3) and mares with RFM (*n* = 3). RNA sequencing was performed with the use of all samples and mRNA expression of chosen genes was validated with Real Time PCR. Analysis of RNA-seq identified 487 differentially expressed genes in the allantochorion and 261 in the endometrium of control and RFM mares (*p* < 0.0001). Within genes that may be important in the release of fetal membranes and were differentially expressed, our report pinpointed *BGN, TIMP1, DRB, CD3E, C3, FCN3, CASP3, BCL2L1.* Gene ontology analysis showed possible processes which were altered in RFM that are apoptosis, inflammatory-related processes, and extracellular matrix metabolism and might be involved in the pathogenesis of RFM. This is the first report on the transcriptome of RFM and physiologically released placenta in mares.

## 1. Introduction

The equine placenta is composed of two parts, maternal that is the endometrium, and fetal that is the allantochorion and amnioallantois [1]. In the last step of parturition, allantochorionic villi slip out of the endometrial crypts, and these two compartments separate, however, the detailed molecular mechanism of the process in mares remains unknown [2,3]. Studies in other species suggest that sterile inflammatory process is the key mechanism of physiological parturition and fetal membrane release [4,5,6,7]. This process is also supported by the remodeling of the placental tissues [8,9]. 

In mares, the failure to expel the allantochorionic membranes within 3 h post-partum is considered as the retention of fetal membranes (RFM) [2,3]. It is one of the most common post-partum diseases and, depending on the horse breed, involves around 10% of the light breed and pony mares and up to 50% of draft and Friesian mares [8]. If not treated, it may have serious life-threatening consequences for a mare, such as laminitis and septicemia [2,3]. 

Pathogenesis of RFM has not been fully elucidated in mares [2,3]. It is suggested that conditions, such as uterine fatigue resulting from low calcium levels, overstretching of the myometrium, various nutritional deficiencies, dystocia, and advanced age of a mare may lead to RFM [2,3]. Among molecular processes which may influence the incidence of RFM, the researchers indicated dysregulation of matrix molecular proteinases (MMPs) and reduced expression of oxytocin receptors [9,10]. Our studies showed that altered expression of inflammatory cytokines IL-1, IL-6, and TNF might be involved in the pathogenesis of this disease [11]. Involvement of neither may be excluded since RFM is suggested to be a multifactorial disease [2,3]. In cows, where RFM also occurs, the alteration in processes, such as apoptosis, inflammatory response, and remodeling of the extracellular matrix, is suggested to contribute to RFM pathogenesis [6,12,13]. Nevertheless, detailed knowledge on the processes undergoing in the equine placenta in the perinatal period and thus possible reasons of RFM remain unknown. 

Comprehensive knowledge of the transcriptome profile allows identifying all expressed genes, biological pathways, and molecular processes differentiating pathological and normal tissues. Such an approach can be a source of valuable information enabling us to investigate the etiology of complex diseases, such as RFM. The goal of the study was to characterize and compare the transcriptomes of the fetal and maternal part of the placenta between mares with RFM and these that expelled fetal membranes physiologically. In addition, molecular pathways, gene ontologies, and biological processes which may be comprised in the placenta of RFM mares will be identified. 

## 2. Materials and Methods

### 2.1. Animals

Samples of the endometrium and the allantochorion were collected from Polish heavy draft mares aged 4–15 years within 2 h of the foal expulsion, as described before [11]. Every mare was monitored by a veterinarian throughout the pregnancy and parturition and only clinically healthy females with the physiological course of parturition were enrolled in the study. Retention of fetal membranes (RFM) was considered as a failure of fetal membrane detachment within 3 h of foal expulsion. Every case of RFM was diagnosed and treated by a veterinarian. Every placenta was examined visually by an experienced veterinarian for the placentitis and samples were taken only from normal placentas [14,15]. Horses were assigned retrospectively to two groups: Mares with RFM (*n* = 3) and control mares (*n* = 3) from which both tissues that are the endometrium and the allantochorion were sampled.

According to the Polish Regulation from 15 January 2015, on the welfare of animals used for experimental or educational purposes and European Directive 2010/63/EU on the protection of animals used for scientific purposes, procedures performed in the project do not need the approval of the Institutional Animal Care and Use Committee, which was confirmed by the Local Ethical Committee at University of Warmia and Mazury in Olsztyn with the decision #LKE.065.07.2019. The owner provided informed consent and voluntarily agreed to sample collection.

### 2.2. RNA Isolation

RNA isolation was performed as described previously [11]. Total RNA was isolated with Total RNA Mini Plus kit (A&A Biotechnology, Gdańsk, Poland) from both the allantochorion and the endometrium according to the manufacturer’s directions. The quantity and quality of mRNA were determined with NanoDrop spectrophotometer (ND200C; Fisher Scientific, Hampton, PA, USA). Additionally, the RNA quality was verified with TapeStation2200 (Agilent, Santa Clara, CA, USA) and RNA ScreenTape (Agilent, Santa Clara, CA, USA) according to protocol. Only RNA samples with RNA integrity values above 7 were selected to further analyses. 

### 2.3. RNA Sequencing 

The RNA-seq was performed for in total 12 samples: Six endometrium and six allantochorion (three samples in each group, RFM and control in each tissue). According to the protocol, 400 ng of total RNA was used to cDNA libraries preparation using TruSeq RNA Library Prep Kit v2 (Illumina, San Diego, CA, USA). The libraries were ligated with different indexes to pool together during sequencing and exclude the possible lane effect. The libraries were amplified in 15 cycles of PCR, quality and quantity were checked using TapeStation 2200 (with Agilent D1000 ScreenTape; Agilent, Santa Clara, CA, USA) and Qubit (with Qubit dsDNA BR Assay Kit; Invitrogen, ThermoFisher Scientific, Whaltam, MA, USA). Next, the libraries were sequenced in 150 pair-end cycles on HiSeq 3000/4000 according to the protocol (Illumina, San Diego, CA, USA). 

### 2.4. NGS Data Analysis and Statistical Approach

In the first step, the reads’ raw quality was analyzed with the FastQC software [16]. After quality control, the filtration of reads was maintained with Flexbar software [17] by removing adapters, reads of phread quality under 30, and minimal read length was set to 50. Then, the mapping procedure was utilized using Tophat software [18], followed by counting of the reads to the gtf annotation file of Equus Caballus 3.0 assemble release v. 97 with the use of htseq-count software [19]. The differential expression analysis was performed with the use of Deseq2 software with the log transformation method [20]. The significance of the output results was set for *p*-value < 0.05 after Benjamini Hochberg correction (adjusted *p*-value). Moreover, to show only genes with high differences fold change (FC) threshold was set ≥ 1.8. Such an approach allowed obtaining a list of differentially expressed genes (DEGs) which were used to determine significantly enriched Gene Ontology terms and pathways. 

Genes identified as significant in both tissues were submitted as genes’ list to DAVID v. 6.8 [21], WebGestalt [22] software and KEGG database in order to find significantly enriched Gene Ontology terms (GO) and pathways. To measure the gene-enrichment in annotation terms, the Fisher’s Exact Test available in DAVID software was applied and the adjusted p-value was shown after Bonferroni correction [23] For WebGestalt software, the Fisher’s Exact Test with Bonferroni correction was used [23].

### 2.5. Validation of NGS Data Using Real Time PCR

The validation of RNA-seq results was performed on nine DEGs. The primers for these genes were designed based on reference sequences using Primer3 web version 4.0.0 (http://bioinfo.ut.ee/primer3/ accession date: 2 October 2020) and are shown in Table S1. As endogenous controls, two genes were selected: *B2M* and *RPL32* [24]. The specificity of the obtained amplicons was checked using AmpliTaq 360 Polymerase (Applied Biosystems, Foster City, CA, USA) and 3% agarose gel electrophoresis. The 250 g of RNA samples were revere transcribed to cDNA using High Capacity RNA to cDNA Kit (Applied Biosystems, Foster City, CA, USA). The gene expression levels were measured in triplicate for each sample using Sensitive RT HS-PCR Mix EvaGreen^®^ (A&A Biotechnology, Gdańsk, Poland) and QuantStudio 7fex (Applied Biosystems, Foster City, CA, USA). The Relative Quantity (RQ) was calculated based on delta delta ct method and according to Pfalff [25]. The comparison between RNA-seq data and qPCR was performed using Pearson correlation (SAS Software v.8.02). Results were considered to be statistically significant for the *p*-value < 0.05. 

## 3. Results

### 3.1. Next Generating Sequencing Basic Characteristic 

The RNA-seq analysis was performed on 12 samples representing the maternal and fetal part of the placenta. After the quality control step, two samples belonging to the control group (one from the endometrium and one from the allantochorion) were removed from further analyses according to a very low mapping rate.

The average number of reads per sample obtained for both tissues was similar (7.5 million and 7.4 million for the allantochorion and the endometrium, respectively). The percentage of reads aligned to the reference genome varied from 75% to 81.7% per sample (average 77.7%, Table S2). 

### 3.2. Identification of Differentially Expressed Genes (DEGs)

**Figure 1:**
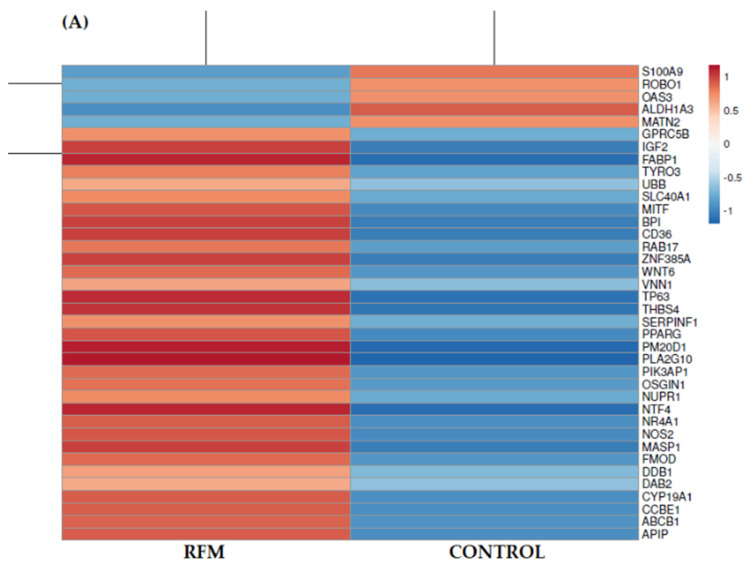

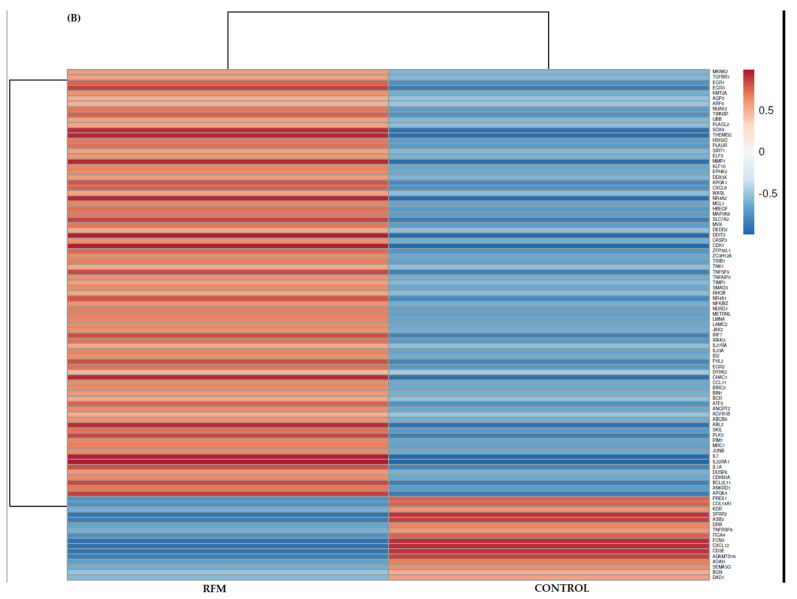
Heatmap showing genes whose expression differs between RFM and control group of mares in the endometrium (**A**) and the allantochorion (**B**). Each row refers to a gene, while columns refer to the investigated groups. Genes with up-regulated expression are shown in red, while with down-regulated in blue. Data were analyzed with the use of ClustVis software [26] and the following parameters: Logarithmic transformation and the Pareto scaling.

Analysis of genes’ expression identified 487 DEGs in the allantochorion from which 109 genes (22 genes with FC > 10) were down-regulated in the RFM group compared to control and 378 (48 with FC > 10) were up-regulated (Figure 1B). From an identified DEGs set, 430 genes were recognized as known genes and 57 represented novel genes. In the endometrium of RFM mares, 28 down-regulated (6 with FC > 10) and 233 up-regulated DEGs (153 genes with FC > 10) were detected, in total 261 (Figure 1A). Out of these genes, 222 DEGs were identified as known genes. The most up-regulated gene in the allantochorion was the early growth response 4 gene (*EGR4*, ENSECAG00000010604), while in the endometrium was long non-coding RNA (*lncRNA*, ENSECAG00000033289) (Table 1). 

### 3.3. Gene Ontology—GO Analysis

All DEGs in the allantochorion and the endometrium were submitted as genes’ list to DAVID v. 6.8 [21] and WebGestalt [22]. In both analyses, output results were considered significant with parameters set to include ≥5 genes with adjusted *p*-value < 0.05. Annotation analysis allowed assigning DEGs from both the endometrium and the allantochorion to gene ontology (GO) terms, which may involve the separation of fetal membranes. The most overrepresented GO for the allantochorion were “biological regulation” and “regulation of biological processes” for which 221 and 214 DEGs were identified, respectively. The most overrepresented GO for the endometrium were “nucleus” with 81 and “cytoplasm” with 67 DEGs, respectively.

**Figure 2:**
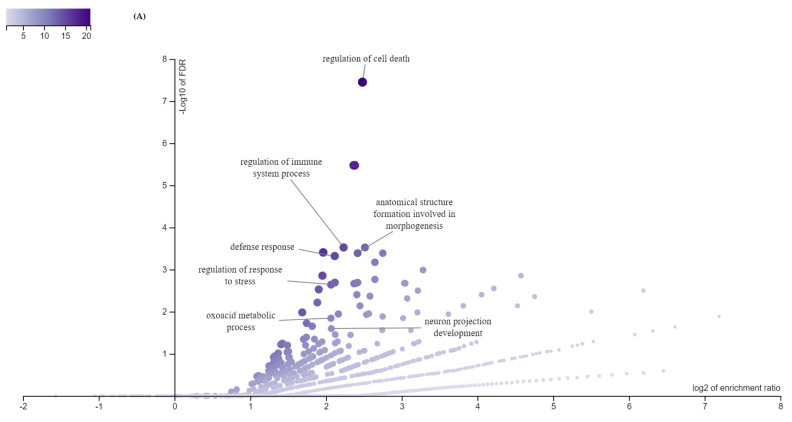

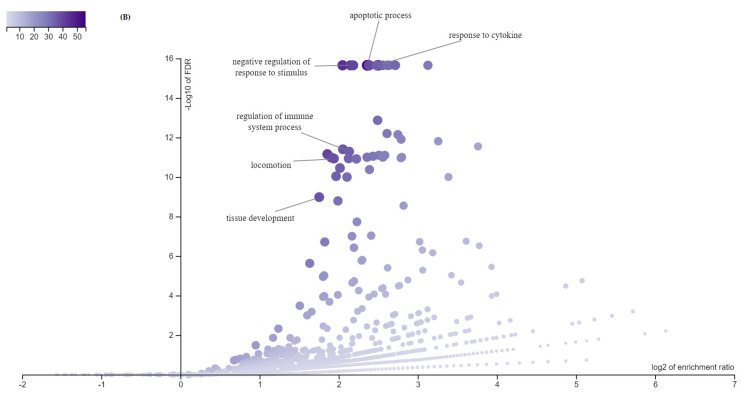
Volcano plot of DEGs expressed in the endometrium (**A**) and the allantochorion (**B**) related to the immune system processes, apoptosis, and extracellular matrix. The size and color of the dots correspond to the size of the gene set. The volcano plot shows −log10 of FDR < 0.05 against log2 enrichment ratio for all the categories. The size and color of the dot are proportional to the size of the category. The position of the dot corresponds to the significance of the category. Analysis was performed in WebGestalt with the use of default settings.

Within significant GOs, those related to apoptosis, the functioning of the immune system, and extracellular matrix remodeling were present both in the endometrium and in the allantochorion. Involved in these processes, DEGs were either up- or down-regulated (Figure 2 and Table S3). 

In the endometrium 15 DEGs and in the allantochorion 22 DEGs were involved in the negative regulation of apoptosis. Except for *CXCL12, KDR, SFRP2* all investigated genes were up-regulated in the placenta of RFM mares. A smaller number of DEGs was associated with the positive regulation of this process, eight genes in the endometrium and 15 in the allantochorion. Two of these genes were down-regulated in the endometrium and two in the allantochorion.

There were 104 DEGs in the allantochorion and 23 in the endometrium in the immune system-related processes. Within these genes, 16 and two were down-regulated in the allantochorion and the endometrium, respectively. The identified DEGs were involved in processes such as the immune system development, response to cytokine, and cellular response to cytokine stimulus.

Regarding extracellular matrix and extracellular matrix organization, there were 11 DEGs (*TIMP1, LAMC2, MMP1, COL14A1, ELF3, SOX9, TGFBR1, SMAD3*) detected in the allantochorion for which three were down-regulated (*SFRP2*, *ADAMTS18*, *BGN*). In turn, in the endometrium tissue, genes representing ECM GO term were identified—*WNT6*, *CCBE1*, *FMOD*, *MATN2*, *SERPINF1*, and *THBS4*.

### 3.4. KEGG Pathways Enrichment Analysis 

Analysis of the biological pathways was performed separately for the endometrium and the allantochorion for all identified DEGs using KEGG database with *Equus caballus* reference with the use of DAVID v. 6.8 software [21]. Parameters were set for pathways including ≥5 genes and for adjusted *p*-value < 0.05. In the allantochorion, three significant pathways that involve at least five DEGs were detected, while in the endometrium, there was one significant pathway that could be potentially involved in the placenta expulsion (Table S3). 

**Figure 3:**
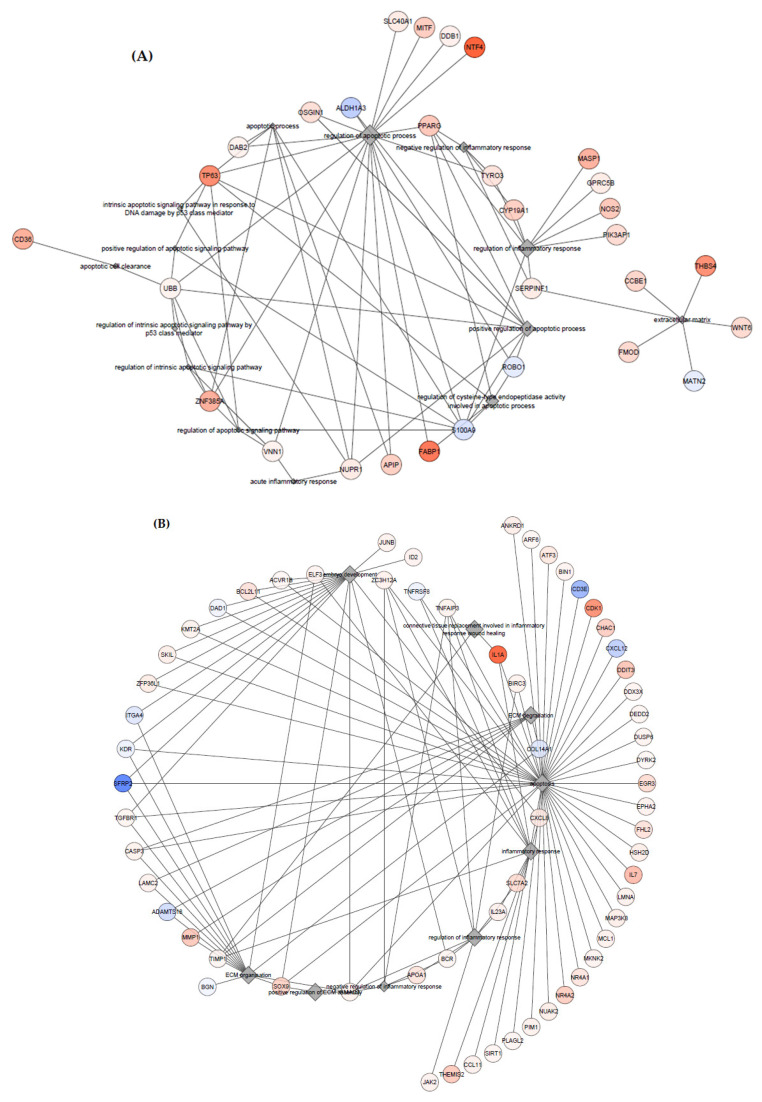
Network of associations between analyzed genes expressed in the endometrium (**A**) and the allantochorion (**B**) and their closest interactions created in Cytoscape v. 3.8.2 with the use of STRING application.

To investigate in more detail the processes undergoing in the RFM and normal placenta, analysis with the use of STRING application in the Cytoscape software v. 3.8.2 was performed for DEGs related to apoptosis, inflammatory, and ECM processes [27]. The analysis was performed separately for the endometrium and the allantochorion. Results demonstrated significant interactions between analyzed DEGs (Figure 3).

### 3.5. Validation Results 

Nine DEGs were used for the validation of RNAseq results (Table 2). Within validated DEGs, seven showed significant correlation coefficients (from 0.4 to 1), which confirmed the RNAseq results’ reliability. The highest similarity of the results was detected for *BGN, CXCL12*, and *lncRNA* genes. Lower correlation coefficients can be associated with additional not yet annotated isoforms. 

## 4. Discussion

### 4.1. Differences in the Transcriptomes of the Allantochorion and Endometrium between RFM and Control Mares

Analysis of the transcriptome of both the allantochorion and the endometrium showed significant differences between RFM and control mares. Different expression profiles suggest that either up- or down-regulated expression of specific genes in the RFM placenta in comparison to the control group may influence its detachment. Moreover, the high throughput RNA-seq method allowed identifying multiple novel genes that were significantly deregulated (with FC > 10) in the placenta of RFM mares. Interestingly, the most up-regulated novel gene in the endometrium of RFM mares was long non-coding RNA (*lncRNA*, ENSECAG00000033289) with FC > 249. LncRNAs function as regulators of gene expression [8,28]. These molecules can control chromatin remodeling mechanisms, sponging miRNAs and act as silencers or enhancers for targeted mRNA [28,29]. Studies in pigs showed that expression of lncRNAs may be tissue-specific and in this species, testes and ovaries were highly enriched in lncRNAs, which might suggest the potential role of these molecules in the regulation of the functioning of the reproductive system [30]. The gene expressed in mares’ placenta in our studies is classified as a “novel gene” in Ensembl database and currently has no orthologues within other species. Hence, further studies are needed to evaluate the possible role of lncRNAs in the placenta of mares. 

Functional analysis of the DEGs pinpoints biological processes which occurred in the equine placenta and genes involved, which may be important for its physiological detachment. Our results showed that genes involved in the biochemical changes within the extracellular matrix and apoptosis were differentially expressed in the placenta of the studied groups of mares. Both processes contribute to the weakening of the attachment between the fetal and maternal part of the placenta [6,9,13,31]. Based on our results, it can be proposed that apoptosis is altered in the placenta of RFM mares. In humans and cows, apoptosis increases as the pregnancy progresses, which contributes to the rupture and later release of membranes during parturition [13,32,33]. Analysis of the retained fetal membranes of cows and mares with the TUNEL method showed an increased number of apoptotic cells in analyzed tissues in comparison to physiologically expelled membranes [13,34]. In cows, apoptosis is required to complete the maturation of the placenta, a process that fosters membrane release [13,33]. In addition, results of studies in women indicate that apoptosis is one of the processes required for the biomechanical weakening of membranes and their further mechanical rupture and separation [35,36]. It can be speculated that similar to these species, apoptosis may be required to support the process of membranes separation in mares. Hence, disturbances in this process may contribute to RFM incidence. Nevertheless, samples of the placenta were taken after the expulsion of the fetus. Impaired apoptosis may also be a consequence of incidents leading to the retention of membranes [13,34]. 

Physical preparation of the fetal membranes for parturition and their detachment also involves remodeling of ECM [30,37,38]. Two of the DEGs involved in the ECM-related GOs, namely *BGN* and *TIMP-1*, may be important in the pathogenesis of RFM. Both molecules are characterized by pleiotropic mechanisms of actions, including ECM turnover [39,40]. In women, when parturition approaches, the expression of BGN in the placenta increases, promoting the disintegration of collagen and hence, weakening of the membranes [41]. Their remodeling is further supported by matrix metalloproteinases (MMPs), which are secreted as inactive enzymes and activated by cleavage of N-terminal propeptide [42]. The activity of MMPs is controlled by various factors, including TIMPs-1 to 4, which are broadly present in the reproductive tract [43]. TIMP-1 is able to inhibit all MMPs, however, with a preference for binding to MMP-1, -2, -3, and -9 and is expressed in the human placenta [40]. Human parturition is characterized by an increase in the active form of MMP-9 and a decrease in active TIMP-1 and -2 what promotes degradation of ECM, cervical ripening, and post-partum involution of the uterus [44]. The results of Rapacz et al. [9] showed that the potential activity of MMP-2 verified by zymography in the allantochorion of RFM mares was lower than in control mares.

Furthermore, expression of TIMP-2 was elevated in the retained placenta of cows, which was suggested to inhibit degradation of ECM and could lead to RFM in this species [6]. Our results correspond to the aforementioned studies and suggest that biomechanical changes within the ECM leading to its degradation may be comprised in RFM mares. In contrast, results of zymography performed in Thoroughbred and pony mares indicate that latent activity of MMP-2 either does not change or even decrease in preparation for labor, whereas MMP-9′s increases only in the amniotic fluid [45]. Clearly, the mechanism of ECM remodeling in mares during physiological and pathological labor is not fully elucidated.

Biglycan (BGN) is not only engaged in maintenance and the assembly of ECM but also various immune-related processes. It is released from ECM of stressed or injured tissues and is one of the danger-associated molecular patterns (DAMPs) [45,46]. In this case, BGN binds and activates toll-like receptors (TLRs)-2 and -4. In turn, it activates signaling pathways p38, NF-κB, ERK and promotes the expression of TNF-α, macrophage inflammatory protein–1 and -2 (MIP-1, -2) [39]. These molecules further activate macrophages, which upon activation, are able to release BGN themselves, which boosts their recruitment and activity [47]. Furthermore, BGN may increase MHC I and II T cells priming via TLR2 and TLR4 pathways and their adaptors MyD88, TRIF, and TIRAP [48]. Increased expression of BGN is suggested to be associated with sterile inflammation, such as in human testes, where this proteoglycan is also expressed [49]. It is feasible to speculate that a similar mechanism takes place during labor where BGN may be released to foster labor-related inflammation. A pilot study by Schoniger et al. [50] showed that mRNA of TLRs-2, -4, and -6 are expressed in the allantochorion of mares. It is possible that decreased expression of BGN in the allantochorion of RFM mares may alter inflammatory-like processes in this group of mares and in consequence, expulsion of fetal membranes.

### 4.2. Inflammatory Processes

Significant KEGG pathways detected in the allantochorion involved only the immune-related processes. A similar result was obtained for GO terms and these include inflammatory response, cytokine production, and interactions, leukocyte migration, and immune system regulation. The majority of genes involved in these processes were up-regulated, which may suggest more profound immune response in the RFM mares or post-partum reaction to retained membranes. Physiological parturition is characterized by sterile inflammation [51] and studies on other species showed increased expression of mediators such as IL1α, MMP-1, and CXCL8, during labor [52], which is in agreement with our results. Notwithstanding, there were few genes involved in the immune processes, whose expression was decreased in the placenta of RFM mares. Among these genes were *DRB* (MHC class II DR-beta chain) and *CD3E*. MHC II is expressed on dendritic cells, macrophages, and B lymphocytes, which are called antigen-presenting cells (APCs), and is required for the presentation of antigens by these cells to CD4+ T lymphocytes [53]. It is also a marker of activation of these cells [54]. Moreover, activated memory CD4+ T cells are also characterized by expression of MHC II [55]. In turn, CD3E is one of subunits building the CD3 module which is essential for surface expression of T cell receptors (TCRs) present on T cells and transduces signals inside the T cell upon an antigen-binding (by TCR) [56]. After binding, the antigen presented by MHC II expressed on APCs by TCR-CD3 complex, activation of T lymphocytes is a key component of the adaptive immune response [57]. Lower expression of MHC II and CD3E may result in the comprised presentation of maternal/fetal antigens [58] and hence, decreased activation of T cells in RFM mares. The adaptive immune response is suggested to be involved in the human labor, including rupture of fetal membranes [59]. Nevertheless, whether this type of immune response is comprised in mares with RFM requires further investigation.

Interestingly, expression of genes engaged in the complement pathway that is *C3* and *FCN3* was decreased in the allantochorion of RFM mares. Complement can be activated via three pathways that are classical, alternative, and lectin-dependent where FCN3 is involved [60]. Its role in parturition has been mostly studied regarding premature parturition and preeclampsia in women [60]. Ficolins, by binding to trophoblast apoptotic cells, activate the lectin-dependent complement pathway and thus participate in removing the apoptotic cells [61]. Complement component 3, the C3, has a central role in the functioning of the complement cascade. Increased levels of C3 are noted in mice with preterm delivery and were associated with degradation of collagen, profound influx of macrophages in the cervix, and increased activity of MMP-9 [62]. It is also suggested that during the cycle, deposition of C3 in the reproductive tract of mice and women might depend on the hormonal status [63].

Moreover, progesterone is able to abrogate complement activation during preterm parturition. Nevertheless, a growing body of evidence from human and mice studies suggests that complement is also one of the components of inflammatory processes present during physiological parturition [64]. Whether activation of complement during physiological parturition is hormone-dependent as it is during cycle remains unknown. However, it is an interesting subject to explore. Based on the expression of *FCN3* and *C3* in the placenta of studied mares, two hypotheses can be inferred. First, the complement activity in the placenta of RFM may be decreased. This, together with the expression pattern of ECM-related DEGs, might contribute to altered remodeling of ECM and, in turn, not efficient loosening of the connection between the fetal and the maternal part of the placenta. Second, lower expression of *FCN3* may be a consequence of differences in apoptosis in the placenta of RFM and control mares. 

All placental samples were collected after the foal’s expulsion, which is the main limitation of the study. Hence, observed differences in the mRNA expression of placental transcriptome may be a cause of the disease, may reflect changes generated by RFM occurrence or may be an additive effect of both. Nevertheless, RFM is a multifactorial disease whose pathogenesis is still not completely elucidated [2,3]. Changes that finally lead to RFM may start at an unknown point of pregnancy, and until the last stage of parturition, it remains unknown whether RFM will occur or not. In addition, taking samples during pregnancy may cause ethical issues. Studies in women investigating the pathogenesis of preterm parturition are based on placental samples taken after parturition and not during pregnancy, even though alterations in the functioning of the placenta may start during pregnancy [65]. We are aware that obtained results should be analyzed with regard to the time of sampling. However, despite this limitation, they bring novel knowledge in the field of equine reproduction and point directions of future research on RFM.

## 5. Conclusions

In conclusion, the present study showed that placental transcriptome differs significantly between mares that retained fetal membranes and those that released them physiologically. There were genes with down-regulated expression among DEGs, such as *BGN, FCN3, C3, CD3E, DRB, CXCL12*, or up-regulated, such as *lncRNAs, TIMP1*, which might be involved in processes present during fetal membranes release. This is the first study comparing transcriptome of normal and retained placenta, and obtained dataset may be used for further studies on processes leading to RFM.

## Figures and Tables

**Table 1:** Top five differentially expressed genes (DEGs) in the endometrium and in the allantochorion with their accession numbers, fold change (FC), and corresponding adjusted *p*-value.

	Gene Symbol	Gene Name	Accession Number	FC	Adjusted *p*-Value
endometrium	*LncRNA*	Long non-coding RNA	ENSECAG00000033289	249	***
	*Novel gene*	Novel gene	ENSECAG00000040328	202,7	***
	*CPA4*	Carboxypeptidase A4	ENSECAG00000018730	179	***
	*LncRNA*	Long non-coding RNA	ENSECAG00000032862	173.8	***
	*GPRIN2*	G protein regulated inducer of neurite outgrowth 2	ENSECAG00000004381	163.7	***
allantochorion	*EGR4*	early growth response 4	ENSECAG00000010604	183	ns ^(0.001)^
	*LRRN4*	leucine rich repeat neuronal 4	ENSECAG00000020607	102.7	****
	*PI16*	peptidase inhibitor 16	ENSECAG00000024845	102	*
	*CPLX1*	complexin 1	ENSECAG00000010131	86	ns ^(0.002)^
	*ALKAL2*	ALK and LTK ligand 2	ENSECAG00000029965	68.9	****

* *p* < 0.05, *** *p* < 0.001, **** *p* < 0.0001, FC—fold change, ns—not significant for adjusted *p*-value, but significant for *p*-value (given in brackets).

**Table 2:** Genes’ expression correlation coefficients between Real Time PCR and RNA-seq data obtained for chosen DEGs.

DEG	Gene Accession Number	Correlation Coefficient
*CASP3*	ENSECAG00000022197	0.4 *
*DRB*	ENSECAG0000001293	0.1
*SMAD3*	ENSECAG00000020259	0.4
*TIMP1*	ENSECAG00000014259	0.6 *
*TNFRSF8*	ENSECAG00000014108	0.2
*CXCL12*	ENSECAG00000019551	1 *
*BGN*	ENSECAG00000018717	0.8 *
*Bcl2l*	ENSECAG00000032970	0.9 *
*lncRNA*	ENSECAG00000033289	0.9 *

Pearson correlation coefficient test, * *p* < 0.05.

## Data Availability

The data presented in this study are available on request from the corresponding author and in the Supplementary Materials.

## Supplementary Materials

The following are available online at https://www.mdpi.com/2076-2615/11/3/675/s1, Table S1. The accession numbers, primers’ sequences and amplicons length of genes validated by Real Time PCR. Table S2. Characteristics of RNA-sequencing results. Table S3. Results of functional annotation analysis of differentially expressed genes (DEGs) in the allantochorion and in the endometrium of mares.
