# Supplementary material for: Transcriptome Profiling of the Retained Fetal Membranes—An Insight in the Possible Pathogenesis of the Disease
Source: Animals (Basel). 2021 Mar 3;11(3):675. doi: 10.3390/ani11030675 (PMC8000898; doi:10.3390/ani11030675)
Supplement: Supplementary file 1 [file animals-11-00675-s001.zip › Table S1.docx]

**Table S1. The accession numbers, primers’ sequences and amplicons length of genes validated by Real Time PCR.**

| ***Gene*** | **Primer sequence (5’–3’), forward/reverse** | **Amplicon length (bp)** | **Accession number** |
| --- | --- | --- | --- |
| *CASP3* | TCCATGGAGAACTCTAAAATCTCA  ATTATACATAACCCCATTTCAGGA | 150 | ENSECAG00000022197 |
| *DRB* | GCGAGTACCGGGCGCTGACCGA  GCTGGGTCTTTGCAGGATAC | 208 | ENSECAG0000001293 |
| *SMAD3* | TGGACGAGCTGGAGAAGG  TCATGGTGGCTGTGTAGGTC | 163 | ENSECAG00000020259 |
| *TIMP1* | CTCCCTGGAACAGTCTGAGC  TCTGGAAGCCCTTGTCAGAG | 245 | ENSECAG00000014259 |
| *TNFRSF8* | ATGGATGCCAAGGTCGTC  ACGATCTGAAGGGCACAGTT | 197 | ENSECAG00000014108 |
| *CXCL12* | GACCCCTGTGCTGGAGAC  TGGGCGCTCAGGTAGTAATC | 155 | ENSECAG00000019551 |
| *BGN* | CATCCATGACAACCGCATC  TCGGAGATTCGCAGGTAGTT | 205 | ENSECAG00000018717 |
| *Bcl2l* | GAGGAGGCGGAGGATGTT  CTGATTGTCGCCACTTAGGC | 196 | ENSECAG00000032970* |
| *lncRNA* | TGGAAGAAACAGTCACAGCAA  TTCCTGACAAAGGTGGGAAG | 231 | ENSECAG00000033289* |

*References:

21. Ahn, K.; Bae, J.-H.; Nam, K.-H.; Lee, C.-E.; Park, K.-D.; Lee, ․ Hak-Kyo; Cho, B.-W.; Kim, H.-S. Identification of Reference Genes for Normalization of Gene Expression in Thoroughbred and Jeju Native Horse(Jeju Pony) Tissues. *Genes Genomics*, **2011**, *33*, 245–250.
