# Supplementary material for: Transcriptome Profiling of the Retained Fetal Membranes—An Insight in the Possible Pathogenesis of the Disease
Source: Animals (Basel). 2021 Mar 3;11(3):675. doi: 10.3390/ani11030675 (PMC8000898; doi:10.3390/ani11030675)
Supplement: Supplementary file 1 [file animals-11-00675-s001.zip › Table S2.docx]

**Table S2. Characteristics of RNA sequencing results.**

| Sample | Number of raw reads | Number of aligned pairs | Percent of concordant pair alignment | Percent of multiple alignments |
| --- | --- | --- | --- | --- |
| 1A | 8515888 | 6759037 | 79.0 | 5.0 |
| 1E | 8030799 | 6442180 | 79.3 | 6.2 |
| 2A | 7376448 | 5807011 | 78.4 | 4.8 |
| 2E | 6090622 | 4694591 | 76.8 | 5.8 |
| 3A | 6490533 | 4904528 | 75.3 | 5.1 |
| 3E | 7425869 | 5635768 | 75.5 | 6.0 |
| 4A | 8076110 | 6625172 | 81.7 | 5.3 |
| 4E | 6941844 | 5357629 | 76.8 | 6.3 |
| 5A | 8986591 | 7130716 | 79.1 | 5.0 |
| 5E | 6924811 | 5220982 | 75.0 | 6.1 |

A – allantochorion; E – endometrium
