# Supplementary material for: Transcriptome Profiling of the Retained Fetal Membranes—An Insight in the Possible Pathogenesis of the Disease
Source: Animals (Basel). 2021 Mar 3;11(3):675. doi: 10.3390/ani11030675 (PMC8000898; doi:10.3390/ani11030675)
Supplement: Supplementary file 1 [file animals-11-00675-s001.zip › Table S3.docx]

**Table S3. Results of functional annotation analysis of differentially expressed genes (DEGs) in the allantochorion and in the endometrium of mares.**

| **ALLANTOCHORION** | | | | | | |
| --- | --- | --- | --- | --- | --- | --- |
| GO term | Number of up-regulated genes | Genes’ names | Numer of downregulated genes | Genes’ names | p value | p-value with Bonferroni correction |
| Enrichment score: 3.62 | | | | | | |
| Apoptotic process GO:0006915 | 43 | *ARF6, MCL1, BCL2L11, CHAC1, DDX3X, DDIT3, EPHA2, MKNK2, NUAK2, PLAGL2, PIM1, SKIL, SMAD3, SOX9, TNFAIP3, ZFP36L1, ATF3, ACVR1B, ANKRD1*  *BIRC3, BIN1, CASP3, CDK1, DEDD2, DAD1, DUSP6, DYRK2, EGR3, FHL2, HSH2D, IL1A, IL7, LMNA, MAP3K8, NR4A1, NR4A2, PLAUR, PLK3, RHOB, SIRT1, TGFBR1, UBB, ZC3H12A* | 5 | *CXCL12*  *CD3E*  *TNFRSF8*  *KDR*  *SFRP2* | 2.8E-7 | 6.0E-5 |
| Apoptotic signaling pathway GO:0097190 | 24 | *MCL1, BCL2L11, CHAC1, DDX3X, DDIT3, EPHA2, MKNK2, PLAGL2, SKIL, SMAD3, TNFAIP3, ATF3, ACVR1B, CDKN1A, DEDD2, DYRK2, IL1A, IL7, LMNA, NR4A2, PLAUR, SIRT1, TGFBR1,*  *UBB* | 4 | *CD3E*  *CXCL12*  *SFRP2*  *TNFRSF8* | 1.7E-6 | 2.1E-4 |
| positive regulation of apoptotic process GO:0043065 | 18 | *MCL1, BCL2L11, DDX3X, DDIT3, PLAGL2, SKIL, SMAD3, ATF3, ANKRD1, BIN1, DEDD2, DUSP6, NR4A1, PLAUR, RHOB, SIRT1, TGFBR1, UBB,ZC3H12A* | 2 | *SFRP2*  *TNFRSF8* | 5.4E-5 | 3.3E-3 |
| regulation of apoptotic signaling pathway GO:2001233 | 15 | *MCL1, BCL2L11, DDX3X, PLAGL2, SKIL, TNFAIP3, ATF3, DEDD2, IL7, LMNA, NR4A2, PLAUR, SIRT1, TGFBR1, UBB* | 2 | *CXCL12*  *SFRP2* | 5.5E-4 | 1.9E-2 |
| intrinsic apoptotic signaling pathway GO:0097193 | 13 | *MCL1, BCL2L11, CHAC1, DDX3X, DDIT3, EPHA2, PLAGL2, SKIL, CDKN1A, DYRK2, PLAUR, SIRT1, UBB* | 1 | *CXCL12* | 8.8E-4 | 2.7E-2 |
| positive regulation of apoptotic signaling pathway GO:2001235 |  | *MCL1, BCL2L11, PLAGL2, SKIL, ATF3, DEDD2, PLAUR, SIRT1, TGFBR1, UBB* |  |  | 1.0E-3 | 2.9E-2 |
| negative regulation of apoptotic process GO:0043066 | 22 | *MCL1, DDX3X, NUAK2, PIM1, SOX9, TNFAIP3, BIRC3, CASP3, CDK1, DAD1, EGR3, FHL2, HSH2D, IL7, LMNA, NR4A1, NR4A2, PLAUR, PLK3, SIRT1, TGFBR1, ZC3H12A* | 3 | *CXCL12*  *KDR*  *SFRP2* | 1.1E-3 | 3.2E-2 |
| positive regulation of intrinsic apoptotic signaling pathway GO:2001244 | 4 | *MCL1, PLAGL2, SKIL, SIRT1, UBB* |  |  | 1.1E-2 | 1.6E-1 |
| negative regulation of intrinsic apoptotic signaling pathway GO:2001243 | 4 | *MCL1, DDX3X, PLAUR, SIRT1* | 1 | *CXCL12* | 8.5E-2 | 5.3E-1 |
| Enrichment score 2.81 | | | | | | |
| immune system process GO:0002376 | 49 | *ABL2, ABCB9, BCL2L11, BCR, CXCL8, DDX3X, EPHA2, JAK2, JUNB, KLF10, MKNK2, NDRG1, SKIL, SMAD3, SOX9, TIPARP, TNFAIP3, WASL , ZFP36L1, ACVR1B, AHSP, ASB2, APOA1, APOA4, AQP3, BIRC3, CASP3, CCL11, CDKN1A, EGR1, EGR3, HSH2D, ID2, IRF7, IL1A, IRAK3, IL23A, IL27RA, IL7, KMT2A, MRC1, SIRT1, SLC7A2, THEMIS2, TGFBR1, TRIB1, TNFSF9, TNK1, ZC3H12A* | 8 | *CXCL12*  *CD3E*  *DRB*  *TNFRSF8*  *FCN3*  *ITGA4*  *PREX1*  *SFRP2* | 3.8E-5 | 2.5E-3 |
| regulation of immune system process GO:0002682 | 28 | *ABCB9, BCR, CXCL8, KLF10, SMAD3, SOX9, TNFAIP3, WASL, ACVR1B, ASB2, APOA1, AQP3, BIRC3, CDKN1A, EGR3, ID2, IRF7, IL1A, IRAK3, IL23A, IL27RA, IL7, SIRT1, SLC7A2, THEMIS2, TRIB1, TNFSF9, ZC3H12A* |  | *CXCL12*  *CD3E*  *FCN3* | 6.7E-3 | 1.1E-1 |
| Enrichment score 2.52 | | | | | | |
| extrinsic apoptotic signaling pathway GO:0097191 | 14 | *MCL1, BCL2L11, DDX3X, MKNK2, SKIL, SMAD3, TNFAIP3, ATF3, ACVR1B, DEDD2, IL1A, IL7, LMNA, TGFBR1* | 1 | *SFRP2* | 2.5E-5 | 1.8E-3 |
| extrinsic apoptotic signaling pathway via death domain receptors GO:0008625 | 5 | *DDX3X, SKIL, TNFAIP3, ATF3, DEDD2* |  |  | 7.4E-3 | 1.2E-1 |
| negative regulation of extrinsic apoptotic signaling pathway GO:2001237 | 5 | *MCL1, TNFAIP3, IL7, LMNA, TGFBR1* | 1 | *SFRP2* | 2.3E-2 | 2.5E-1 |
| Enrichment score 2.18 | | | | | | |
| inflammatory response GO:0006954 | 17 | *BCR, CXCL8, ELF3, EPHA2, JAK2, NFKBIZ, SMAD3, TNFAIP3, AOAH, APOA1, CCL11, IL1A, IL23A, METRNL, MVK, SLC7A2, ZC3H12A* | 2 | *CXCL12*  *TNFRSF8* | 7.4E-4 | 2.4E-2 |
| negative regulation of inflammatory response GO:0050728 | 8 | *BCR, SMAD3, TNFAIP3, AOAH, APOA1, METRNL, MVK, ZC3H12A* |  |  | 1.6E-3 | 3.9E-2 |
| Enrichment score 2.03 | | | | | | |
| response to cytokine GO:0034097 | 20 | *MCL1, CXCL8, JAK2, JUNB, SOX9, TIMP1, ANKRD1, APOA1, CCL11, EGR1, IRF7, IL1A, IRAK3, IL22RA1, MRC1, SIRT1, ZC3H12A* | 2 | *CXCL12*  *BGN* | 1.7E-3 | 4.2E-2 |
| cytokine-mediated signaling pathway GO:0019221 | 10 | *CXCL8, JAK2, APOA1, CCL11, EGR1, IRF7, IL1A , IRAK3, IL22RA1, SIRT1* | 2 | *CXCL12*  *BGN* | 1.3E-2 | 1.7E-1 |
| response to tumor necrosis factor GO:0034612 | 7 | *CXCL8, JAK2, ANKRD1, APOA1, CCL11, SIRT1, ZC3H12A* |  |  | 2.1E-2 | 2.3E-1 |
| cellular response to interleukin-1 GO:0071347 | 8 | *CXCL8, SOX9, ANKRD1, CCL11, EGR1, IL1A, IRAK3, ZC3H12A* |  |  | 2.6E-4 | 1.0E-2 |
| Enrichment score 1.75 | | | | | | |
| regulation of leukocyte activation GO:0002694 | 10 | *BCR, TNFAIP3, CDKN1A, EGR3, ID2, IL23A, IL27RA, IL7, SLC7A2, TNFSF9* | 1 | *CD3E* | 1.4E-1 | 6.7E-1 |
| Enrichment score 1.6 | | | | | | |
| innate immune response GO:0045087 | 12 | *ABL2, DDX3X, JAK2, TNFAIP3, APOA4, BIRC3, CCL11, IRF7, IRAK3, IL23A, MRC1, TNK1* | 1 | *FCN3* | 8.0E-2 | 5.1E-1 |
| Enrichment score 1.17 | | | | | | |
| negative regulation of inflammatory response GO:0050728 | 7 | *BCR, SMAD3, TNFAIP3, AOAH, APOA1, METRNL, ZC3H12A* |  |  | 1.6E-3 | 3.9E-2 |
| negative regulation of cytokine production GO:0001818 | 6 | *EPHA2, TNFAIP3, APOA1, IRAK3, IL23A, ZC3H12A* |  |  | 2.2E-1 | 7.9E-1 |
| Enrichment score 0.98 | | | | | | |
| leukocyte chemotaxis GO:0030595 | 3 | *CXCL8, CCL11, IL23A* | 2 | *CXCL12*  *PREX1* | 3.4E-1 | 9.0E-1 |
| Enrichment score 0.97 | | | | | | |
| response to insulin GO:0032868 | 5 | *RAB31, CRY2, EGR1, SESN2, SIRT1* |  |  | 2.8E-1 | 8.5E-1 |
| glucose homeostasis GO:0042593 | 3 | *CRY2, SESN2, SIRT1* |  |  | 7.7E-1 | 1.0E0 |
| Enrichment score 0.95 | | | | | | |
| neutrophil migration GO:1990266 | 4 | *CXCL8, CCL11, IL1A, IL23A* |  | *PREX1* | 5.5E-2 | 4.2E-1 |
| leukocyte migration GO:0050900 | 6 | *BCR, CXCL8, WASL, CCL11, IL1A, IL23A* | 3 | *CXCL12*  *ITGA4*  *PREX1* | 7.6E-2 | 5.0E-1 |
| granulocyte migration GO:0097530 | 4 | *CXCL8, CCL11, IL1A, IL23A* | 1 | *PREX1* | 9.2E-2 | 5.5E-1 |
| chemotaxis  GO:0006935 | 8 | *CXCL8, EPHA2, ANGPT2, CCL11, EGR2, EGR3, HBEGF, IL23A* | 4 | *CXCL12*  *KDR*  *PREX1*  *SEMA3G* | 2.0E-1 | 7.6E-1 |
| Enrichment score 0.93 | | | | | | |
| leukocyte differentiation GO:0002521 | 12 | *EPHA2, JUNB, KLF10, ZFP36L1, EGR1, EGR3, ID2, IL23A, IL7, SIRT1, TRIB1, TNFSF9* | 2 | *CD3E*  *PREX1* | 5.9E-2 | 4.3E-1 |
| leukocyte activation GO:0045321 | 16 | *BCR, CXCL8, NDRG1, SMAD3, TNFAIP3, ZFP36L1, CDKN1A, EGR1, EGR3, HSH2D, ID2, IL23A, IL27RA, IL7, SLC7A2, TNFSF9* | 2 | *CD3E*  *PREX1* | 6.1E-2 | 4.4E-1 |
| leukocyte cell-cell adhesion GO:0007159 | 9 | *ZFP36L1, APOA4, EGR1, EGR3, HSH2D, IL23A, IL7, TNFSF9, SMAD3* | 4 | *CXCL12*  *CD3E*  *ITGA4*  *PREX1* | 9.2E-2 | 5.5E-1 |
| Enrichment score 0.85 | | | | | | |
| extracellular matrix organization GO:0030198 | 4 | *ELF3, SOX9, TGFBR1, SMAD3* |  | *SFRP2* | 4.7E-1 | 9.5E-1 |
| Enrichment score 0.74 | | | | | | |
| tumor necrosis factor production GO:0032640 | 4 | *TNFAIP3, IRAK3, IL23A, ZC3H12A* | 1 | *TNFRSF8* | 8.5E-2 | 5.3E-1 |
| regulation of tumor necrosis factor superfamily cytokine production GO:1903555 | 4 | *TNFAIP3, IRAK3, IL23A, ZC3H12A* | 1 | *TNFRSF8* | 9.2E-2 | 5.5E-1 |
| Enrichment score 0.14 | | | | | | |
| extracellular matrix GO:0031012 | 4 | *TIMP1, LAMC2, MMP1, COL14A1* | 2 | *ADAMTS18*  *BGN* | 7.2E-1 | 9.9E-1 |
|  |  |  |  |  |  |  |
| KEGG pathway | Number of up-regulated genes | Genes’ names | Numer of downregulated genes | Genes’ names | p value | p-value with Bonferroni correction |
|  |  | Enrichment score 0.98 |  |  |  |  |
| cytokine-cytokine receptor interaction ecb04060 | 12 | *CXCL8, ACVR1B, CCL11, IL1A, IL10RA, IL11, IL22RA1, IL23A,*  *IL7, LIF, TGFBR1, TNFSF9* | 2 | *CXCL12*  *TNFRSF8* | 7.1E-4 | 7.3E-2 |
| chemokine signaling pathway ecb04062 | 5 | *CXCL8, JAK2, WASL, CCL11, PIK3R3* | 3 | *CXCL12, PREX1, GNG11* |  |  |
| Enrichment score 0.93 | | | | | | |
| TGF-beta signaling pathway ecb04350 | 5 | *SMAD3, TGIF1, ACVR1B,*  *ID2, TGFBR1* |  |  | 1.2E-1 | 5.2E-1 |
| ***ENDOMETRIUM*** | | | | | | |
| GO term | Number of up-regulated genes | Genes’ names | Numer of downregulated genes | Genes’ names | p value | p-value with Bonferroni correction |
| Enrichment score 1.3 | | | | | | |
| regulation of apoptotic process GO:0042981 | 16 | *APIP, DAB2, TYRO3, DDB1, FABP1, MITF, NTF4, NUPR1, NR4A1,*  *OSGIN1, PPARG, SLC40A1, TP63, UBB, VNN1, ZNF385A* | 2 | *ALDH1A3,*  *ROBO1* | 2.1E-2 | 7.9E-1 |
| negative regulation of cell death GO:0060548 | 13 | *APIP, DAB2, TYRO3, DDB1, FABP1, NTF4,*  *NR4A1, PM20D1, PRKD1,*  *SLC40A1, TP63, VNN1,*  *ZNF385A* |  |  | 3.2E-2 | 8.3E-1 |
| positive regulation of cell death GO:0010942 | 6 | *NUPR1, NR4A1, OSGIN1, PPARG, TP63,*  *UBB* | 2 | *ALDH1A3*  *ROBO1* | 8.7E-2 | 8.8E-1 |
| apoptotic process GO:0006915 | 16 | *APIP, DAB2, TYRO3, DDB1, FABP1, NTF4, NUPR1, NR4A1, OSGIN1, PPARG, PDK1,*  *SLC40A1, TP63, UBB, VNN1, ZNF385A* | 2 | *ALDH1A3*  *ROBO1* | 5.0E-2 | 8.5E-1 |
| positive regulation of apoptotic process GO:0043065 | 6 | *NUPR1, NR4A1, OSGIN1, PPARG,*  *TP63, UBB* | 2 | *ALDH1A3*  *ROBO1* | 7.6E-2 | 8.8E-1 |
| negative regulation of apoptotic process GO:0043066 | 11 | *APIP, DAB2, TYRO3, DDB1, FABP1, NTF4,*  *NR4A1, SLC40A1, TP63,*  *VNN1, ZNF385A* |  |  | 8.0E-2 | 8.8E-1 |
| Enrichment score 1.04 | | | | | | |
| cellular response to nitrogen compound GO:1901699 | 7 | *ACTN2, EIF4EBP1, FOXO4,*  *IGF2, NR4A1, PPARG SESN1* |  |  | 9.0E-2 | 8.8E-1 |
| Enrichment score 0.83 | | | | | | |
| regulation of inflammatory response GO:0050727 | 5 | *GPRC5B, TYRO3, CYP19A1,*  *NOS2, PIK3AP1* | 1 | *S100A9* | 5.6E-2 | 8.6E-1 |
| immune system process GO:0002376 | 21 | *ABCB1, CD36, GPRC5B,*  *RAB17, TYRO3, BPI, CYP19A1, FABP1, IGF2,*  *MASP1, MITF, NOS2, PM20D1, PPARG, PIK3AP1, PLA2G10,*  *SLC40A1, THBS4, VNN1,*  *ZNF385A* | 2 | *OAS3*  *S100A9* | 1.6E-1 | 9.2E-1 |
| innate immune response GO:0045087 | 6 | *TYRO3, MASP1, NOS2,*  *PPARG, PIK3AP1, VNN1* | 1 | *S100A9* | 1.8E-1 | 9.2E-1 |
| inflammatory response GO:0006954 | 6 | *GPRC5B, TYRO3, CYP19A1,*  *NOS2, NUPR1, VNN1* |  |  | 3.4E-1 | 9.6E-1 |
| negative regulation of immune system process GO:0002683 | 5 | *TYRO3, BPI, CYP19A1,*  *PPARG, PIK3AP1* |  |  | 3.4E-1 | 9.6E-1 |
| positive regulation of immune response GO:0050778 | 5 | *CD36, GPRC5B, TYRO3, MASP1, PIK3AP1* |  |  | 4.4E-1 | 9.8E-1 |
| Enrichment score 0.67 | | | | | | |
| intrinsic apoptotic signaling pathway GO:0097193 | 6 | *NUPR1, PDK1, TP63, UBB,*  *VNN1, ZNF385A* |  |  | 8.4E-2 | 8.8E-1 |
| Enrichment score 0.52 | | | | | | |
| extracellular matrix GO:0031012 | 6 | *WNT6, CCBE1, FMOD,*  *MATN2, SERPINF1, THBS4* |  |  | 2.4E-1 | 9.8E-1 |
| Enrichment score 0.38 | | | | | | |
| regulation of cytokine production GO:0001817 | 5 | *CD36, GPRC5B, BPI, CCBE1, NOS2* |  |  | 6.5E-1 | 1.0E0 |
| Enrichment score 0.18 | | | | | | |
| positive regulation of immune system process GO:0002684 | 9 | *ABCB1, CD36, GPRC5B,*  *TYRO3, IGF2, MASP1, PIK3AP1, THBS4, VNN1* |  |  | 2.2E-1 | 9.3E-1 |
| regulation of leukocyte activation GO:0002694 | 5 | *TYRO3, BPI, IGF2, PLA2G10, VNN1* |  |  | 4.3E-1 | 9.7E-1 |
| KEGG pathway | Number of up-regulated genes | Genes’ names | Numer of downregulated genes | Genes’ names | p value | p-value with Bonferroni correction |
| Enrichment score 1.18 | | | | | | |
| metabolic pathways ecb01100 | 20 | *BDH1, PTS, APIP, POLR2I,*  *ALDH3B1, AGPS, B4GALNT1, CYP17A1, CYP2C92, CYP19A1,*  *ETNPPL, EXTL1, GAL3ST1,*  *HSD3B2, NOS2, PLA2G10 , PTGES, PDXK, RGN,*  *TYRP1* | 3 | *ALDH1A3*  *ALDOC*  *SMPD1* | 1.7E-2 | 5.6E-1 |
| Enrichment score 1.56 | | | | | | |
| ovarian steroidogenesis ecb04913 | 5 | *CYP17A1, CYP19A1, HSD3B2, LHB, STAR* |  |  | 2.0E-3 | 2.4E-1 |
